# WKYMVm ameliorates obesity by improving lipid metabolism and leptin signalling

**DOI:** 10.1111/jcmm.17910

**Published:** 2023-08-21

**Authors:** Ji Hyeon Kang, Hyung Sik Kim, Seon Hyang Park, Ye Seon Kim, Yoe‐Sik Bae

**Affiliations:** ^1^ Department of Biological Sciences Sungkyunkwan University Suwon Korea; ^2^ Convergence Research Center for Energy and Environmental Sciences Sungkyunkwan University Suwon Korea; ^3^ Present address: Department of Target Discovery LG Life Science Seoul Korea

**Keywords:** appetite, leptin, lipid metabolism, obesity, WKYMVm

## Abstract

Obesity is a metabolic disorder that results from an imbalance of energy intake and consumption. As low‐grade chronic inflammation caused by obesity can lead to various complications, it is important to develop effective treatments against obesity. In this study, we investigate the effects of WKYMVm, a strong anti‐inflammatory agent, against obesity. Administration of WKYMVm into high fat diet (HFD)‐induced obese mice significantly attenuated body weight gain, food intake and increased insulin sensitivity. HFD‐induced hepatic steatosis and adipose tissue hypertrophy were also markedly ameliorated by WKYMVm. During the maturation of adipocytes, WKYMVm improves lipid metabolism by increasing lipolysis, adipogenesis, mitochondrial biogenesis and fat browning. WKYMVm administration also elicited a decrease in leptin levels, but an increase in leptin sensitivity via regulation of hypothalamic endoplasmic reticulum stress and the leptin receptor cascade. Taken together, our results show that WKYMVm ameliorates obesity by improving lipid metabolism and leptin signalling, suggesting that WKYMVm can be a useful molecule for the development of anti‐obesity agents.

## 1 INTRODUCTION

Obesity, which is defined as excessive body weight with an abnormal accumulation of adipose tissue, is caused by various factors, including eating habits, poor exercise patterns and genetic influences.^1^, ^2^ The World Health Organization defines being overweight as a body mass index of 25.0–29.9 and obesity as a body mass index of 30 and above. More than 2 billion people worldwide are overweight or obese, and the numbers have almost tripled since the 1980s.^3^

During the pathological progression of obesity, changes in energy metabolism and the recruitment of immune cells are closely associated with adipose tissue plasticity.^4^, ^5^, ^6^ Excessive energy resulting from imbalance of energy intake and consumption is stored as triglycerides in adipose tissue, causing adipocyte hypertrophy and increasing adipokine production.^1^, ^2^ Various immune cells, including inflammatory macrophages and neutrophils, accumulate in obese adipose tissue and secrete pro‐inflammatory cytokines.^6^ These events elicit low levels of systemic inflammation without infection, causing tissue damage and organ dysfunction. As a result, obesity remains one of the most prominent risk factors for chronic diseases such as Type 2 diabetes,^7^ fatty liver disease^8^ and cardiovascular disease.^9^ Therefore, it is important for human health to identify molecules that control obesity by focusing on the inflammatory response.

Another significant characteristic feature of obesity is high circulating levels of leptin, a phenomenon termed hyperleptinaemia.^10^ Leptin is a long‐term regulator of energy balance which is produced by adipose tissue and carries information on energy deposits to the brain.^11^ In lean mice, circulating leptin crosses the blood–brain barrier and binds to LepRb in the hypothalamic arcuate nucleus, activating the JAK2‐STA3 pathway.^11^, ^12^ During appetite regulation, leptin increases a ‘non‐eating’ peptide, proopiomelanocortin (POMC), but suppresses the production of an ‘eating’ peptide, neuropeptide Y (NPY). LepRb is also involved in the reception of inhibitory signals, which can be derived from multiple negative feedback loops, including suppressor of cytokine signalling 3 (SOCS3) and protein tyrosine phosphatase 1B (PTP1B), to prevent excessive physiological responses.^12^ In obese mice, increased circulating leptin levels and endoplasmic reticulum stress in the hypothalamus induce excessive activation of the negative feedback system, which eventually leads to a disrupted LepRb signalling cascade and eventual resistance to leptin.^11^, ^13^ For this reason, approaches to induce a partial decrease in circulating leptin and simultaneous increase in leptin sensitivity are being studied to replace other treatments for obesity with adverse effects.

Trp‐Lys‐Tyr‐Met‐Val‐D‐Met‐NH_2_ (WKYMVm), an immune stimulating peptide, is a surrogate agonist for the formyl peptide receptor (FPR) family.^14^ Previous reports demonstrated that WKYMVm administration elicits beneficial outcomes against several inflammatory diseases including polymicrobial sepsis, ulcerative colitis, noneosinophilic asthma and hypoxia‐induced lung injury.^15^ Mechanistically, WKYMVm acts as an anti‐inflammatory agent by blocking the production of inflammatory cytokines such as TNF‐α, IL‐1β, IL‐6 and CCL2 induced by LPS in mouse neutrophils and mouse macrophage cells.^16^, ^17^ WKYMVm also improves insulin sensitivity by sensitising the insulin pathway of metabolic tissue in diabetic mice and the palmitic acid‐induced insulin resistance model of L6 myotubes in an FPR2‐dependent manner.^18^ A previous study reported that WKYMVm regulates the production of inflammatory cytokines in white adipose tissue (WAT) by downregulating TNF‐α and IL‐1β.^18^ In this study, we found that WKYMVm has a therapeutic effect on the chronic inflammation environment of obesity. We also examined the mechanism involved in the WKYMVm‐induced anti‐obesity effect by focusing on the regulation of lipid metabolism and food intake.

## 2 MATERIALS AND METHODS

### 2.1 High fat diet (HFD)‐induced obese mouse model

All animal experiments were approved by the Institutional Review Committee for Animal Care and Use at Sungkyunkwan University. Male wild‐type C57BL/6N mice (8‐week‐old, 21 ± 2 g) were purchased from Orient Bio. In order to reduce the weight variables between individuals, two mice were raised in each cage. The mice were housed on a 12 h light/dark cycle and had free access to food and water at 22°C. For the obese mouse model, C57BL/6N mice (8‐week‐old) were fed with a HFD (60% fat as kcal; Research Diets) for 6 or 10 weeks.

### 2.2 WKYMVm injection in obese mouse model

WKYMVm peptide was synthesized by Anygen. After 4 weeks of HFD feeding, obese mice received subcutaneous injections of vehicle (distilled water [DW]) or WKYMVm (8 mg/kg) once every 2 days. During the 2 or 5 weeks of injection, the body weight (g) and food intake (g/week) of the mice were recorded every week. The change in weight (%) was calculated as the (final weight − initial weight)/initial weight × 100. Organs including adipose tissue, liver and brain were harvested and weighed after mice were sacrificed.

### 2.3 Measurement of body mass

Body composition (fat and lean body masses) was measured by 1H magnetic resonance spectroscopy (Bruker BioSpin).

### 2.4 Measurement of blood glucose and insulin levels

For the oral‐glucose tolerance test (O‐GTT), the obese mice were fasted overnight for 10 h, and then injected orally with 2 g/kg glucose. Blood was drawn from the tail vein at specified time intervals, and glucose levels were measured using a glucometer. For the insulin tolerance test (ITT), the mice were fasted overnight for 10 h and then injected with 0.75 U/kg body weight of human insulin via an intraperitoneal injection. Blood glucose levels were monitored at specified time intervals. Plasma insulin levels were determined using ELISA kits manufactured by ALPCO.

### 2.5 Tissue histology

Tissues were fixed in 10% (v/v) neutral buffered formalin (NBF) for 5 days at 37°C and then embedded in paraffin. Tissue blocks were cut into 4‐μm‐thick slices and deparaffinized, rehydrated, and stained with haematoxylin and eosin for histological analysis. Lipid areas (%) and adipocyte areas (μm^2^ × 1000) were quantified using NIH ImageJ software.

### 2.6 Culture of primary adipocytes

Primary adipocytes were isolated from inguinal white adipose tissue (ingWAT) of C57BL/6N (6‐week‐old) mice. Briefly, isolated ingWAT was digested with 1.5 mg/mL collagenase in 10% FBS DMEM, and then mature adipocytes and connective tissues were separated from the cell pellet via centrifugation. The remaining stromal vascular cells were then resuspended in DMEM/F12 with 10% FBS and 1% penicillin/streptomycin (P/S) and seeded in 6‐well plates for adipogenic differentiation. To induce differentiation, 90%–95% confluent cells were treated with a medium containing 5% FBS, 10 μg/mL insulin, 120 μM indomethacin, 0.2 μM dexamethasone, 0.5 mM 3‐isobutyl‐1‐methylxanthine, 1 nM 3,3′,5‐triiodo‐l‐thyronine and 1% P/S for 3 days. After this period, the medium was replaced with maintenance medium containing 10% FBS, 10 μg/mL insulin and 1% P/S for an additional 2 days. Finally, the cells were maintained in DMEM/F12 with 10% FBS and 1% P/S. To test the effects of WKYMVm on adipocyte differentiation and lipid accumulation, the adipocytes were treated with vehicle or 1 μM WKYMVm (Anygen) during the induction stage for 7 days or the maintenance stage for 2 days.

### 2.7 Quantitative RT‐PCR (qRT‐PCR)

Total RNA was isolated by TRIzol reagent (Life Technology) according to the manufacturer's protocol. Isolated RNA was used to synthesize cDNA using a Maxime RT PreMix Kit (Intron) Quantitative polymerase chain reaction (qPCR) was performed with a Roter‐Gene SYBERGreen PCR Kit (QIAGEN) and gene‐specific primers: *fpr1*‐forward, 5′‐CCTTGGCTTTCTTCAACAGC‐3′; *fpr1*‐reverse, 5′‐ GCCCGTTCTTTACATTGCAT‐3′; *fpr2*‐forward, 5′‐GTCAAGATCAACAGAAGAAACC‐3′; *fpr2*‐reverse, 5′‐GGGCTCTCTCAAGACTATAAGG‐3′; *pparg2*‐forward, 5′‐GCATGGTGCCTTCGCTGA‐3′; *pparg2*‐reverse, 5′‐ TGGCATCTCTGTGTCAACCATG‐3′; *ap2*‐forward, GGAAGCTTGTCTCCAGTGAA‐3′; *ap2*‐reverse, 5′‐ GCGGTGATTTCATCGAATTC‐3′; *lpl*‐forward, 5′‐ CTTCTTGATTTACACGGAGGT‐3′; *lpl*‐reverse, 5′‐ATGGCATTTCACAAACACTG‐3′; *atgl*‐forward, 5′‐ TGTGGCCTCATTCCTCCTAC‐3′; *atgl*‐reverse, 5′‐TCGTGGATGTTGGTGGAGCT‐3′; *mgl*‐forward, 5′‐CATTGCTCGCTCCACTCTT‐3′; *mgl*‐reverse, 5′‐ATGGTCCTGATTTCACCTCTG −3′; *hsl*‐forward, 5′‐GCTGGGCTGTCAAGCACTGT‐3′; *hsl*‐reverse, 5′‐GTAACTGGGTAGGCTGCCAT‐3′; *nrf1*‐forward, 5′‐TGGAACAGCAGTGGCAAGATCTCA‐3′; *nrf1*‐reverse, 5′‐GGCACTGTACAGGATTTCACTTGC‐3′; t*fam1*‐forward, 5′‐GCTCAGAACCCAGATGCAAAA‐3′; t*fam1*‐reverse, 5′‐GCCACTCCGCCCTATAAGC‐3′; *pgc1a*‐forward, 5′‐AGCACACGTTTATTCACGGGT‐3′; *pgcla*‐reverse, 5′‐ GCCCCCAAGTCCTCACATG‐3′; *ucp1*‐forward, 5′‐ACTGCCACACCTCCAGTCATT‐3′; *ucp1*‐reverse, 5′‐CTTTGCCTCACTCAGGATTGG‐3′; *obrb*‐forward, 5′‐GCATGCAGAATCAGTGATATTTGG‐3′; *obrb‐*reverse, 5′‐CAAGCTGTATCGACACTGATTTCTTC‐3′; *ptp1b*‐forward, 5′‐TGGATCTCAGACATTCCACACTCAC‐3′; *ptp1b‐*reverse, 5′‐AGCTGCCTTGCTTCCAGTCC‐3′; *ptprj*‐forward, 5′‐CACAGCTGAGATAGCCGAGAACA‐3′; *ptprj‐*reverse, 5′‐GTCGAATGGGTCTGGACTGAAAG‐3′; *pomc*‐forward, 5′‐ CCCTCCTGCTTCAGACCTC‐3′; *pomc‐*reverse, 5′‐CGTTGCCAGGAAACACGG‐3′; *npy*‐forward, 5′‐CTGACCCTCGCTCTATCTCTGC‐3′; *npy‐*reverse, 5′‐CCATCACCACATGGAAGGGTCT‐3′; *xbp1s*‐forward, 5′‐ACACGCTTGGGAATGGACAC‐3′; *xbp1s‐*reverse, 5′‐CCATGGGAAGATGTTCTGGG‐3′; *chop*‐forward, 5′‐GCATGAAGGAGAAGGAGCAG‐3′; *chop‐*reverse, 5′‐ CTTCCGGAGAGACAGACAGG‐3′; *erdj4*‐forward, 5′‐CCCCAGTGTCAAACTGTACCAG‐3′; *erdj4‐*reverse, 5′‐AGCGTTTCCAATTTTCCATAAATT‐3′; *cebpa*‐forward, 5′‐GATTCCTGCTTCCTCTCGGG‐3′; *cebpa*‐reverse, 5′‐TCCCCAACACCTAAGTCCCT‐3′; *srebp1c*‐forward, 5′‐ACTTTTCCTTAACGTGGGCCT‐3′; *srebp1c*‐reverse, 5′‐ TGAGCTGGAGCATGTCTTCG‐3′; *tfrap2b*‐reverse, 5′‐GTGCCGGTCCTCATAGATGTC‐3′; *tfrap2b*‐forward, 5′‐AGCGTCGGATTTGGTGTGT‐3′; *Gapdh*‐forward, 5′‐TCCACCACCCTGTTGCTGTA‐3′; and *Gapdh*‐reverse, 5′‐AATGTGTCCGTCGTGGAT‐CT‐3′. For qPCR, 55 PCR cycles were performed in three steps including denaturation (95°C, 30 s), annealing (60°C, 30 s) and extension (72°C, 1 min). Relative gene expression levels were normalized to *Gapdh* expression levels.

### 2.8 Measurement of leptin levels

To measure the levels of leptin, serum or epidermal white adipose tissue (eWAT) were collected from HFD‐induced obese mice at 7 weeks. Culture medium of primary adipocytes in the absence or presence of WKYMVm was collected. Leptin levels were measured using an enzyme‐linked immunosorbent assay (ELISA) kit (eBioscience Inc.) with antibody pairs, following the manufacturer's instructions.

### 2.9 Western blot analysis

The harvested hypothalamus was lysed in radioimmunoprecipitation assay buffer (RIPA buffer) supplemented with protease inhibitor cocktails (Sigma) at a cold temperature. Extracted proteins were separated by sodium dodecyl sulphate‐polyacrylamide gel electrophoresis (SDS‐PAGE) (8%–15% gel) and transferred to a nitrocellulose membrane (Cytiva). The levels of p‐Stat3 (1:1000, Y705, #9131), p‐Akt (1:1000, S473, #9271), p‐PERK (1:1000, T980, #3179), SOCS3 (1:1000, #2932) were measured. To confirm equal loading, β‐actin was detected using an anti‐β‐actin antibody. All antibodies used for Western blot analysis were purchased from Cell Signaling Technology.

### 2.10 Statistical analysis

All results were evaluated via GraphPad Prism software. Results are expressed as the mean ± SEM (standard error of the mean). Statistical analyses were performed using Student's *t*‐test or analysis of variance (anova). *p* ≤ 0.05 was considered statistically significant.

## 3 RESULTS

### 3.1 WKYMVm ameliorates HFD‐induced obesity

**FIGURE 1:**
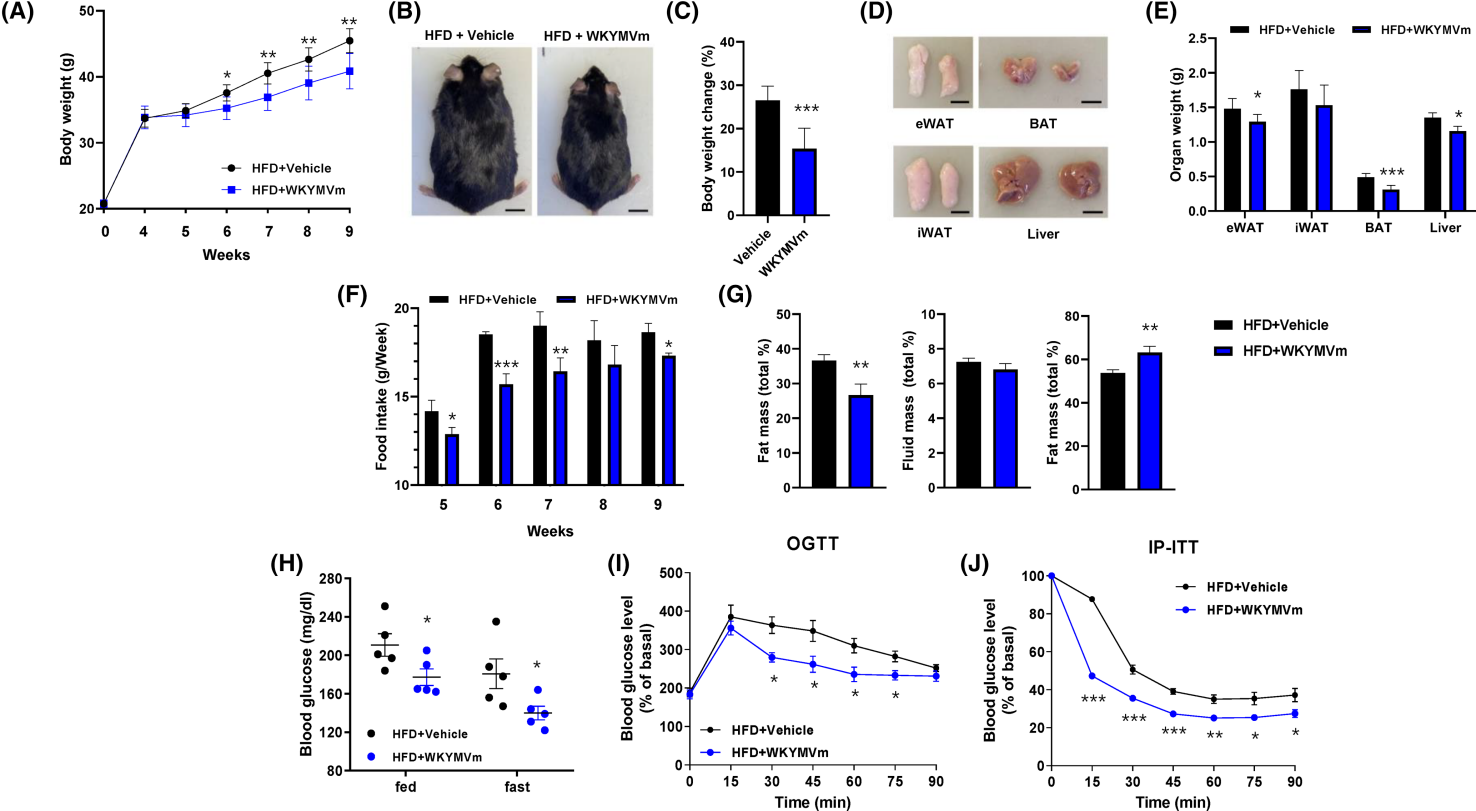
WKYMVm ameliorates HFD‐induced obesity. (A) After housing C57BL/6 mice with a HFD for 4 weeks, 8 mg/kg of WKYMVm was subcutaneously injected every 2 days for 5 weeks. (A–C) Body weight curve (A), representative images (B) and body weight change (%) (C) of vehicle‐ or WKYMVm‐injected obese mice. (D, E) Representative images (D) and weight (E) of adipose tissue and liver from vehicle‐ or WKYMVm‐injected obese mice (*n* = 5/group). Scale bars, 1 cm. (F) Food intake (g/Week) from vehicle‐ or WKYMVm‐injected obese mice. (G) Fat mass, fluid mass and lean mass (%) of vehicle‐ or WKYMVm‐injected obese mice. (H) Blood glucose concentrations from HFD‐induced obese mice for 12 weeks. (I, J) Blood glucose concentrations during O‐GTT (I) and IP‐ITT (J) in fasted HFD‐induced control and WKYMVm‐injected mice for 12 weeks. The data are presented as the mean ± SEM. *p < 0.05; **p < 0.01; ***p < 0.001 by two‐way anova (A, I and J) or Student's *t*‐test (C, E, F, G, and H). All data are representative of three independent experiments (*n* = 5 ~ 6/group, A–J). HFD, high fat diet.

To investigate the effects of WKYMVm in obese mice, C57BL/6 WT mice were fed a HFD for 4 weeks and subsequently 8 mg/kg of WKYMVm was subcutaneously injected every 2 days for 5 weeks. As shown in Figure 1A,B, administration of WKYMVm significantly attenuated weight gain compared to the vehicle group. Notably, the final weight gain relative to the initial weight was ~25% and ~15% in the vehicle‐ or WKYMVm‐administered group, respectively (Figure 1C). The size and weight of metabolic tissues including adipose tissue and liver were also significantly decreased by WKYMVm administration (Figure 1D,E). WKYMVm administration also decreases food intake compared to the vehicle (Figure 1F). We further examined the effects of WKYMVm on several types of body masses and found that WKYMVm administration reduces fat mass and increases lean mass without affecting fluid mass (Figure 1G).

Abnormality in blood sugar (glucose) is a major feature of HFD‐induced obese mice.^2^ Our finding that WKYMVm decreases whole body weight as well as fat mass led us to investigate the effects of WKYMVm on blood glucose concentration. WKYMVm administration decreases blood glucose levels regardless of feeding or fasting compared to the vehicle control (Figure 1H). Consistently, significant improvements in glucose metabolism and insulin sensitivity were induced by WKYMVm (Figure 1I,J). Collectively, our results indicate that WKYMVm has beneficial effects to prevent HFD‐induced obesity and glucose intolerance.

### 3.2 WKYMVm improves HFD‐induced histological damage in the liver and adipose tissues

**FIGURE 2:**
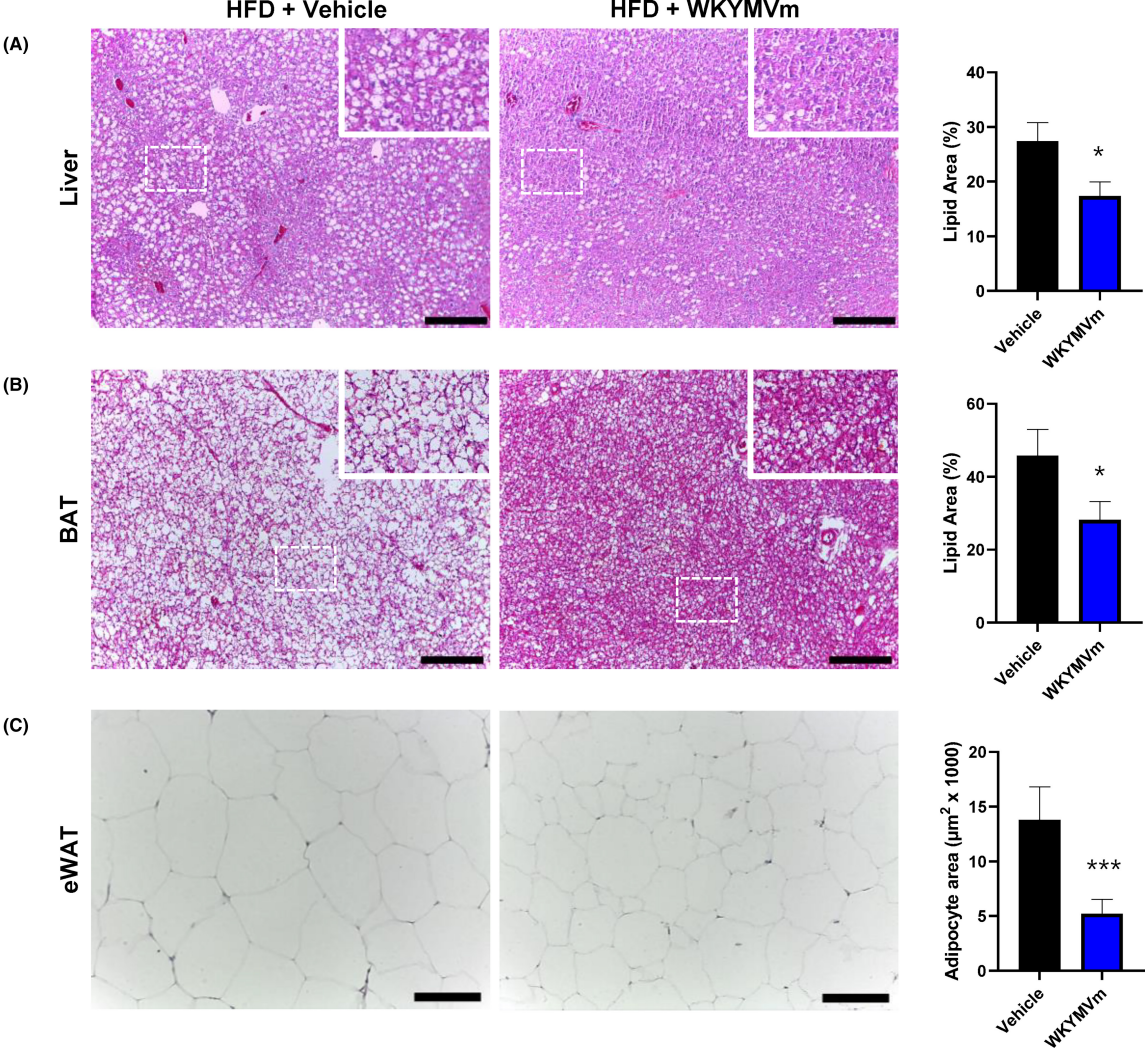
WKYMVm improves HFD‐induced histological damage. (A–C) Representative images of haematoxylin and eosin staining of liver (A), brown adipose tissue (BAT) (B) and epidermal white adipose tissue (eWAT) (C) from vehicle‐ or WKYMVm‐injected obese mice. Scale bar, 50 μm. Magnification, ×100 for (A) and (B); ×200 for (C). The data are presented as the mean ± SEM. *p < 0.05; ***p < 0.001 by Student's *t*‐test, and all data are representative of three independent experiments (*n* = 3/group, A–C). HFD, high fat diet.

As the size and weight of metabolic tissues were significantly reduced by WKYMVm administration (Figure 1D,E), we performed histological analysis with haematoxylin and eosin staining. Similar to a previous report,^5^ HFD feeding causes hepatic lipid accumulation and adipose tissue hypertrophy (Figure 2A–C). However, administration of WKYMVm markedly reduced fat droplets in the liver. Quantitative analysis showed that WKYMVm significantly decreased the lipid area in the liver of HFD‐induced obese mice (Figure 2A). The results indicate that WKYMVm may attenuate hepatic steatosis in obese mice. HFD feeding markedly induced lipid accumulation in brown adipose tissue (BAT), but WKYMVm‐administered mice showed significantly reduced lipid accumulation in the BAT (Figure 2B). Adipocyte hypertrophy is commonly observed in HFD‐fed mice.^2^ We also found that HFD feeding induces adipocyte hypertrophy in eWAT, which was markedly attenuated by WKYMVm administration (Figure 2C left). Quantitative analysis shows that the area of white adipocytes was decreased from 13,000 μm^2^ to 6000 μm^2^ by WKYMVm administration in eWAT (Figure 2C right).

### 3.3 WKYMVm improves lipid metabolism in adipose tissue

**FIGURE 3:**
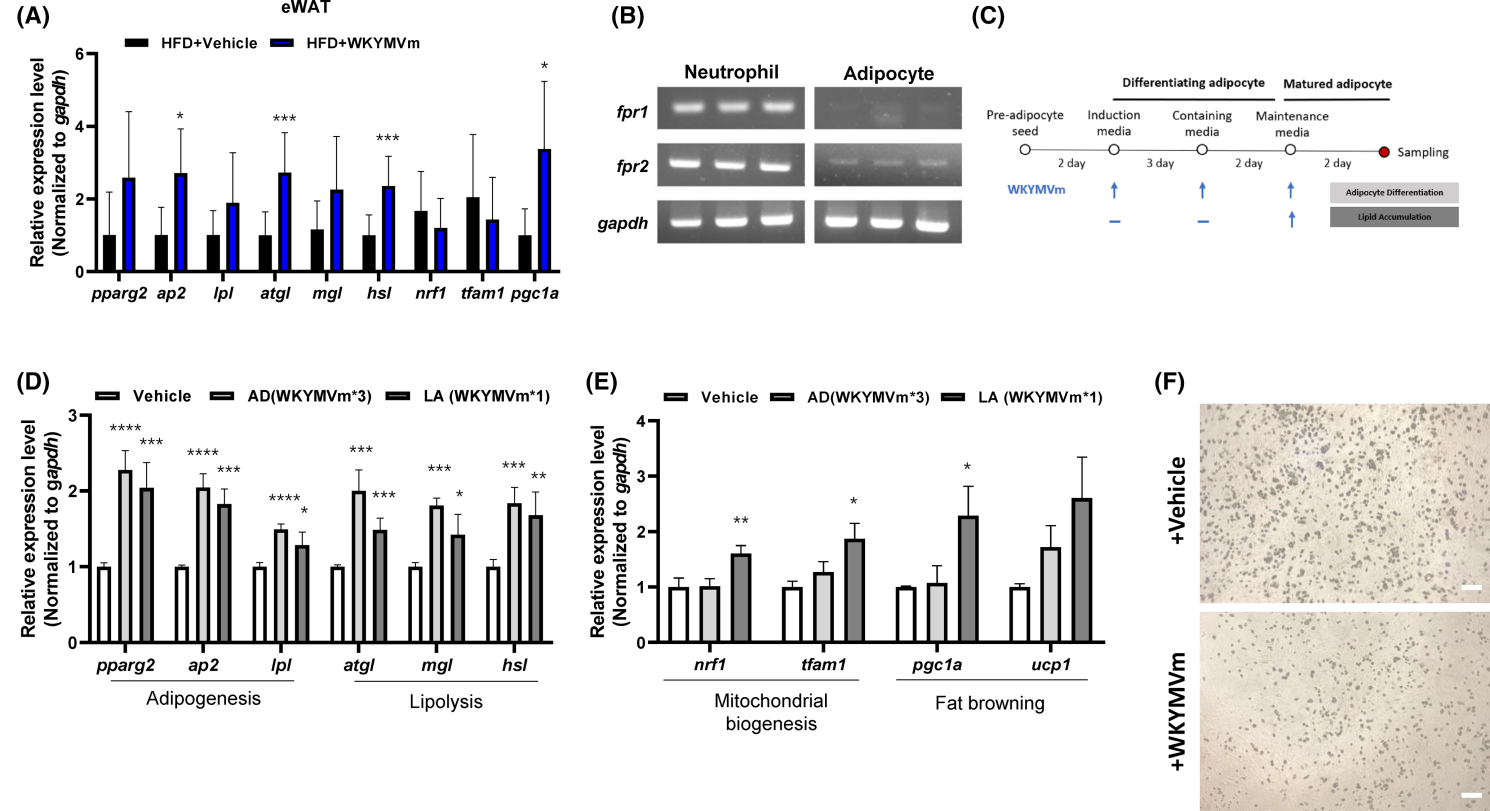
WKYMVm improves lipid metabolism in adipose tissue. (A) Relative mRNA expression of the thermogenic genes in eWAT from HFD‐induced control and WKYMVm‐injected mice for 12 weeks. (B) The expression of *fpr1*, *fpr2* and *gapdh* in neutrophils and adipocytes. (C) Schematic illustration of in vitro experiments to investigate the effects of WKYMVm in primary adipocytes. (D, E) mRNA expression measured by RT‐qPCR. Adipogenesis, lipolysis, mitochondrial biogenesis and fat browning‐related gene expression in adipocytes. (F) Representative images of mature adipocytes in the absence or presence of WKYMVm. Scale bar, 20 μm. Magnification, ×100. The data are presented as the mean ± SEM. *p < 0.05; **p < 0.01; ***p < 0.001 by Student's *t*‐test, and all data are representative of three independent experiments (*n* = 3 ~ 5/group, A–E). AD, adipocyte differentiation; HFD, high fat diet; LA, lipid accumulation.

As we found that WKYMVm administration reduces not only the weight but also the histological damage of adipose tissues and liver (Figures 1D,E, 2A–C), we examined the effect of WKYMVm on lipid metabolism in HFD‐induced obese mice. Administration of WKYMVm significantly increases the expression of genes associated with lipolysis including *atgl*, *mgl* and *hsl*. Some genes related to adipogenesis such as *pparg2*, *ap2* and *lpl* were also increased by WKYMVm in eWAT (Figure 3A). We then investigated if WKYMVm directly regulates lipid metabolism in adipocytes. For this, we examined if primary adipocytes express FPR members (*fpr1* and *fpr2*). By PCR analysis, we found that primary adipocytes express both *fpr1* and *fpr2*, although at a lower level compared to neutrophils (Figure 3B). Addition of WKYMVm during the differentiation of adipocytes as depicted in Figure 3C upregulates several genes associated with adipogenesis. As shown in Figure 3D, the expression of *ppar2*, *ap2* and *lpl* were significantly increased by WKYMVm. This upregulation of adipogenesis‐associated genes was induced when WKYMVm was added to mature adipocytes as well as pre‐adipocytes (Figure 3C,D). We also examined the effects of WKYMVm on the expression of several genes associated with mitochondrial biogenesis. Stimulation of mature adipocytes with WKYMVm significantly increases the expression of *nrf1* and *tfam* which play key roles in mitochondria biogenesis (Figure 3E). Two crucial genes involved in fat browning, *pgc1* and *ucp1*, are markedly upregulated by WKYMVm in mature adipocytes (Figure 3E). However, WKYMVm did not affect the expression of these genes associated with mitochondria biogenesis and fat browning in pre‐adipocytes (Figure 3E). As adipocytes mature, lipid droplets increase in size and number. However, upon WKYMVm treatment, the size and number of these lipid droplets significantly decreased (Figure 3F). These results align with the regulation of lipid metabolism‐related gene expression induced by WKYMVm treatment. Collectively, these results suggest that WKYMVm may directly act on adipose tissue and significantly improve lipid metabolism.

### 3.4 WKYMVm improves leptin signalling in the hypothalamus

**FIGURE 4:**
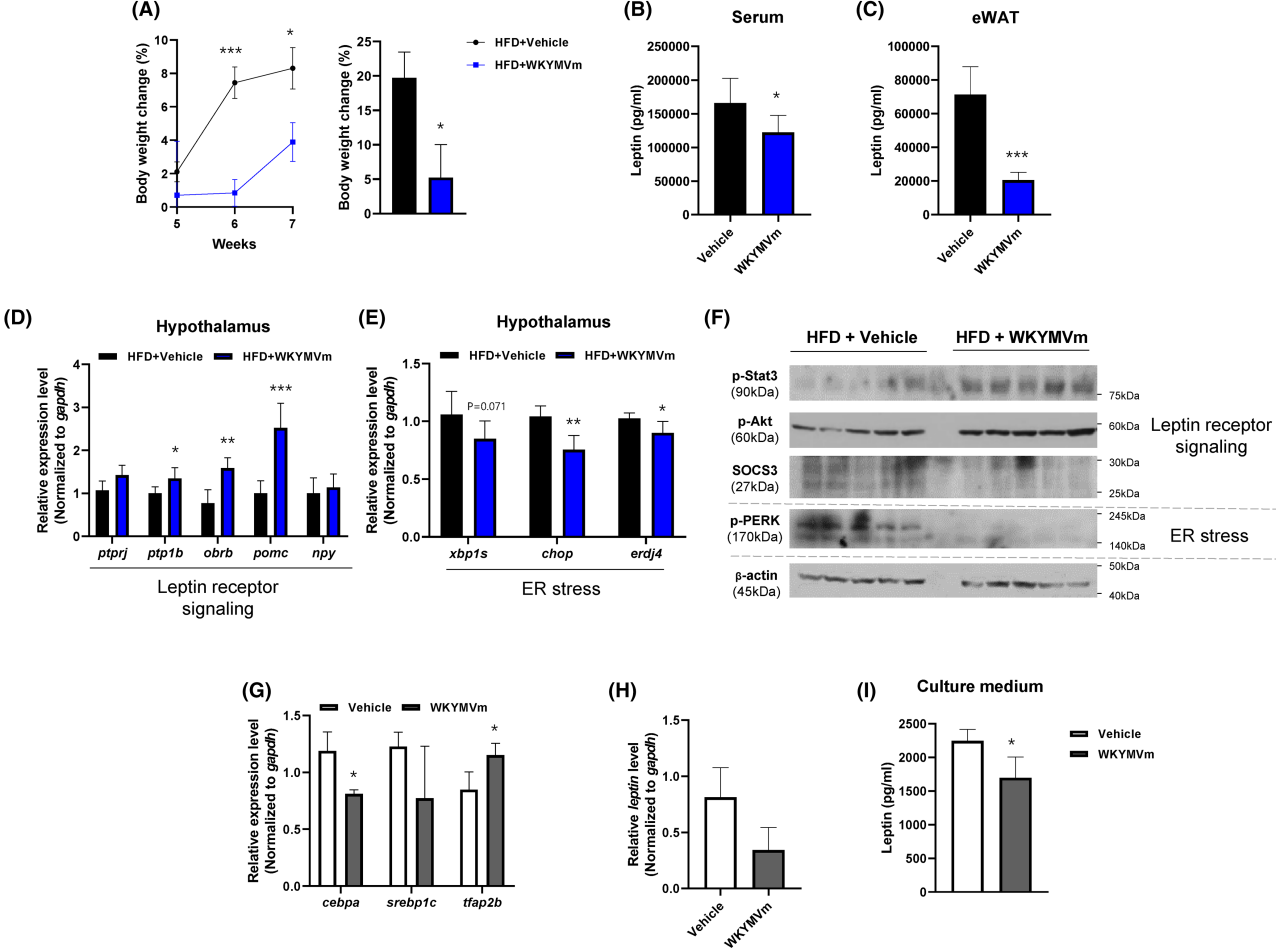
WKYMVm improves leptin signalling in the hypothalamus. (A) Body weight change (%) of vehicle‐ or WKYMVm‐injected obese mice over 2 weeks. (B, C) Leptin concentration of serum (B) and eWAT (C) from vehicle‐ or WKYMVm‐injected obese mice. (D, E) mRNA expression measured by RT‐qPCR. Leptin signalling (D) and ER stress (E)‐related gene expression in the hypothalamus of vehicle‐ or WKYMVm‐injected obese mice. (F) Representative Western blot images of phospho‐PERK, phospho‐Stat3, phospho‐Akt, SOCS3 and β‐actin in the hypothalamus of vehicle‐ or WKYMVm‐injected obese mice. (G, H) mRNA expression measured by RT‐qPCR. Leptin synthesis‐related gene expression in mature adipocytes. (I) The concentration of leptin in the adipocyte culture medium both with and without WKYMVm treatment. The data are presented as the mean ± SEM. *p < 0.05; **p < 0.01; ***p < 0.001 by two‐way anova (A [left]) or Student's *t*‐test (A [right], B–E, and G‐I). All data are representative of three independent experiments (*n* = 3 ~ 5/group, A–I). HFD, high fat diet.

Leptin, a hormone released from adipose tissues, has been reported to play key roles to maintain normal body weight.^10^ However, leptin resistance contributes to diet‐induced obesity, showing a decreased ability of leptin to suppress food intake or increase body energy use.^12^, ^19^ Considering that WKYMVm inhibits food intake in HFD‐induced obesity (Figure 1F), we checked whether WKYMVm attenuates leptin resistance. Similar to the results in Figure 1, WKYMVm administration significantly attenuates body weight increases at 6–7 weeks after the start of HFD feeding (Figure 4A). We found that WKYMVm administration significantly reduces leptin levels in the serum and eWAT at 7 weeks in HFD‐induced obese mice (Figure 4B,C). Because excessive circulating leptin is strongly associated with defective leptin signalling and impaired POMC processing,^12^ we next investigated the leptin signalling cascade. Interestingly, the WKYMVm‐administrated group maintained higher levels of *ptp1b*, *obrb* and *pomc* mRNA in the hypothalamus compared to the vehicle group (Figure 4D). Mechanistically, ER stress is one of the major factors that mediate leptin resistance.^13^ Therefore, we investigated the effects of WKYMVm on ER stress in HFD‐induced obese mice. As expected, the expression of ER stress‐associated genes (x*bp1*, *chop* and *erdf4*) was decreased by WKYMVm administration in hypothalamus (Figure 4E). Moreover, phosphorylation of PERK, an ER‐resident protein mediating the ER stress response, was increased in the hypothalamus of HFD‐fed mice. However, WKYMVm administration markedly reduces phosphorylated PERK levels in the hypothalamus (Figure 4F). A previous report demonstrated that STAT3 signalling plays a crucial role in regulating both ER stress and leptin action in hypothalamus.^20^, ^21^ We also found that HFD‐fed mice showed very low levels of phosphorylated STAT3, which was markedly increased by WKYMVm administration (Figure 4F). WKYMVm administration also increases the levels of phospho‐Akt, which promotes POMC production in hypothalamus (Figure 4F). Furthermore, the levels of SOCS3, a negative regulator of leptin signalling, were decreased by WKYMVm administration (Figure 4F). Considering that leptin is primarily produced by primary adipocytes of WAT,^22^ we conducted an investigation to assess the impact of WKYMVm on leptin synthesis in these adipocytes. Upon exposing mature adipocytes to WKYMVm stimulation, we observed a downregulation in the expression of *cebpa*, a gene involved in leptin synthesis. Conversely, the expression of *tfap2b*, a gene that inhibits leptin synthesis, was significantly upregulated in the adipocytes (Figure 4G). Additionally, WKYMVm treatment led to a decrease in the expression of leptin at both mRNA and protein levels (Figure 4H,I). Collectively, our results suggest that WKYMVm improves leptin sensitivity by comprehensive modulation of leptin secretion and signalling in adipose tissue‐hypothalamus axis.

## 4 DISCUSSION

Current treatments for obesity include improved eating habits and physical activity, and in severe cases, drug or surgical treatment can be used.^23^ Most drugs on the market aim to reduce 5%–10% of obese patients' weight. Pentamidine, orlistat and GLP‐1 receptor agonists are representative drugs, but they have various side effects such as neurological and digestive problems.^23^, ^24^ Therefore, new treatment approaches are continuously being explored. In this study, we found that an FPR agonist, WKYMVm, has effective anti‐obesity activity in an HFD‐fed mouse model (Figure 1A–E). WKYMVm administration also improves glucose metabolism and insulin sensitivity in the mice model (Figure 1H–J). HFD‐induced tissue damages such as hepatic steatosis and adipose tissue hypertrophy were also attenuated by WKYMVm (Figure 2). Mechanistically, WKYMVm administration improves leptin resistance as well as lipid metabolism (Figures 3 and 4). WKYMVm is a well known agonist for the FPR family, which are G‐protein‐coupled receptors that are expressed in various immune cells and organs.^25^ Many previous reports demonstrated that activation of FPR members by diverse agonists elicits several cellular responses including chemotactic migration, phagocytosis and superoxide anion production.^25^, ^26^ Some reports revealed a relationship between FPR ligands and obesity. N‐Formyl methionyl‐leucyl‐phenylalanine and serum amyloid A are measured at very high levels in the blood of obese mice and obese patients, and promote insulin resistance as well as weight gain.^27^, ^28^ Conversely, resolvin D1, D2 and lipoxin A4 are known to attenuate obesity, by reducing chronic inflammation by polarising M1 to M2.^29^, ^30^ However, it remains unclear how FPR regulates obesity. Here we found that WKYMVm administration reduces food intake by improving leptin resistance (Figure 1F and 4F). Leptin is thought to be an attractive treatment for obesity due to its role in controlling appetite.^10^ However, these clinical approaches failed in most patients due to ‘leptin resistance’, which leads to interference in leptin receptor signalling.^10^, ^12^, ^19^ Recently, a major conceptual shift in leptin therapy was proposed. Rather than treating obese patients with exogenous leptin, an approach to increase leptin sensitivity by inducing a partial reduction in circulating leptin was proposed.^13^, ^31^ In this study, we found that WKYMVm administration decreases leptin production from eWAT of HFD‐fed obese mice and mature adipocytes (Figure 4C,I). This reduction in leptin production coincides with changes in the expression of genes involved in the synthesis of leptin, including *cebpa* and *tfap2b* (Figure 4G). The reduction in total leptin production resulted in the alleviation of leptin resistance in the hypothalamus. WKYMVm administration elicited increased p‐Stat3 and p‐Akt, which are important players in the leptin receptor signalling pathways, and maintained high expression levels of leptin receptor signalling‐related genes including *obrb* and *pomc*. WKYMVm blocks inhibitory leptin signals, which are regulated by various negative feedback loops, including SOCS3 (Figure 4F). Remarkably, WKYMVm administration also led to a significant reduction in hypothalamic ER stress, a major contributor to leptin resistance. This effect was evident from the notable decrease in both ER stress‐related gene and protein expressions in response to WKYMVm treatment (Figure 4E,F). Obesity is also known to induce hypothalamic inflammation, disrupting metabolic homeostasis.^32^ While the expression of Fpr1 and Fpr2 in the hypothalamus is lower compared to other tissues (data not shown), it will be necessary to investigate the potential pathway of obesity reduction through inhibiting the inflammatory response in the hypothalamus, as WKYMVm demonstrates anti‐inflammatory characteristics. Taking these findings together, we propose that WKYMVm effectively alleviates the ‘overload’ of leptin signalling and can exert significant control over excessive appetite induced by obesity.

As WKYMVm binds to FPR members and FPR members are mainly expressed in innate immune cells, previous studies on the effects and functional roles of FPR members have focused on neutrophils, monocytes and macrophages.^25^, ^33^ However, FPR members are also expressed in non‐immune cells, such as adipose‐derived stem cells and mesenchymal stem cells.^34^, ^35^ Activation of FPR1 signalling negatively regulates the differentiation of mesenchymal stem cells into adipocytes. In this study, we found that WKYMVm administration elicits reduced adipose tissue weight and hepatic steatosis/white adipose tissue hypertrophy in HFD‐induced obese mice (Figure 1D,E, and Figure 2). We also observed that WKYMVm modulates lipid metabolism in vivo as well as in vitro, showing increased expression of genes associated with adipogenesis and lipolysis (Figure 3A,D). Our findings that primary adipocytes express both *fpr1* and *fpr2*, and direct addition of WKYMVm into primary adipocytes effectively modulates the expression of genes in lipid metabolism suggest that WKYMVm may act on primary adipocytes, resulting in the improvement of lipid metabolism in obesity. Obesity‐induced mitochondrial stress in adipocytes can cause decreased glucose homeostasis, adipogenesis and free fatty acid oxidation which can induce insulin resistance and Type 2 diabetes.^36^, ^37^ During the maturation of primary adipocytes, WKYMVm treatment markedly enhanced mitochondrial biogenesis and fat browning (Figure 3D,E). Recently, stimulation of lipolysis has become a therapeutic interest for obesity.^38^, ^39^ Although the role of lipase is considered to worsen obesity in the gastrointestinal tract, it is advantageous to hydrolyse triglycerides to lower lipids in adipocytes. In adipose tissue, WKYMVm not only increases lipolysis but also improves the activity of mitochondria that can oxidize free fatty acid, so it could be utilized as a therapeutic agent with a new approach.

In conclusion, in this study, we demonstrated that administration of the FPR agonist WKYMVm effectively elicits anti‐obesity activity in an HFD‐induced model. Through analysis of biochemical signalling pathways, we propose that WKYMVm may improve leptin resistance, leading to reduced food intake directed by the hypothalamus. Gene expression analysis reveals that WKYMVm also directly improves lipid metabolism. Collectively, WKYMVm and its target FPR members are potentially useful material and promising targets to control obesity.

## AUTHOR CONTRIBUTIONS

**Ji Hyeon Kang:** Conceptualization (lead); data curation (lead); formal analysis (lead); investigation (lead); methodology (lead); validation (lead); visualization (lead); writing – original draft (lead); writing – review and editing (supporting). **Hyung Sik Kim:** Conceptualization (supporting); methodology (supporting). **Seon Hyang Park:** Data curation (supporting); investigation (supporting). **Ye Seon Kim:** Data curation (supporting); investigation (supporting). **Yoe‐Sik Bae:** Conceptualization (lead); funding acquisition (lead); project administration (lead); supervision (lead); writing – original draft (supporting); writing – review and editing (lead).

## CONFLICT OF INTEREST STATEMENT

The authors have no conflicts of interest to disclose.

## Data Availability

The data that support the findings of this study are available from the corresponding author upon reasonable request.
